# The influence of self-pollen deposition on female reproductive success in a self-incompatible plant, *Akebia quinata*

**DOI:** 10.3389/fpls.2022.935217

**Published:** 2022-08-10

**Authors:** Chun-Hui Wang, Ting-Ting Zou, Wei-Qi Liu, Xiao-Fan Wang

**Affiliations:** ^1^College of Life Sciences, Wuhan University, Wuhan, China; ^2^Ecology and Environment Monitoring and Scientific Research Center, Yangtze Basin Ecology and Environment Administration, Ministry of Ecology and Environment of the People’s Republic of China, Wuhan, China

**Keywords:** *Akebia quinata*, geitonogamy, self-incompatible, self-pollen, reproductive success

## Abstract

Geitonogamy is inevitable in hermaphrodite and monecious. Even for self-incompatible species, the negative effects of self-pollen are unavoidable when geitonogamous or self-mating occurs. However, the influence of self-pollen on consecutive development of flowers (e.g., fruiting and seeding) was seldom evaluated. Here, the self-incompatible monecious species, *Akebia quinata*, was used to estimate the influence of self-pollen deposition. We evaluated the extent of pollen limitation and geitonogamous mating under natural conditions by count of stigmatic pollen load and pollen tracking experiment. Hand pollination with different amount and combinations of self vs. cross pollen grains was applied to detect the response of fruit and seed set. The results showed that geitonogamy and pollen limitation occurred under natural conditions in *A. quinata*. Carpel numbers, ratio of self- and cross-pollen, and the interactive effect of ratio of self- and cross-pollen and total mixed pollen numbers, and not total pollen grain number, determined the effect of self-pollen on female reproductive success. The effect of self-pollen depended on its intensity. In general, the transfer of self-pollen significantly affected young fruit set. However, a little self-pollen together with cross-pollen did not reduce young fruit production. Although self-incompatible plants have evolved physiological mechanisms that reduce self-fertilization, our results provide new insights into the effects of self-pollen and the adaptive significance of self-incompatible monecious species.

## Introduction

The sexual system of plants determines the genetic variation and reproductive success while simultaneously affecting the evolutionary processes of flowering plants (Charlesworth and Wright, 2001; Queenborough et al., 2009). Hermaphroditism occurs as the fundamental sexual condition in 75% of all flowering plants (De Jong et al., 1993; Campbell, 2000). In such condition, male and female organs can use the same pollination advertising and rewarding strategies to ensure reproductive success (e.g., nectar, petals, and sepals). Thus, the benefits and effectiveness of single-visit pollination are evident (Charnov et al., 1976). Additionally, approximately 6 and 5% of flowering plants are diecious and monecious, respectively (Renner and Ricklefs, 1995; Diggle et al., 2011). Diecious plants are usually large size and pollinated by abiotic vectors, such as the wind or water, whereas monecious species show separate male and female flowers on the same individual, whereby intra-flower self-pollination can be effectively prevented (Barrett, 1998).

The movement of a pollinator from one flower to another in the same hermaphrodite of monecious plant is inevitable, especially in species with large floral displays (Harder and Barrett, 1995). This phenomenon is the reason for deposition of self-pollen or geitonogamy, whose occurrence which is actually quite common in many plant species, may increase in a plant with many open flowers (Dudash, 1991; De Jong et al., 1992). However, geitonogamy has been largely ignored in pollination biology, and there are very few studies on its quantitative importance and impact on the evolution of mating systems (Charnov, 1982; Geber, 1985; Hessing, 1988; De Jong and Klinkhamer, 1989). The effect of geitonogamy-induced self-pollination on subsequent development of flowers (e.g., fruiting and seeding) has rarely been evaluated, regardless of self-incompatibility.

Self-fertilization affects reproductive success negatively by causing inbreeding depression and interference with fruit or seed set (De Jong et al., 1992, 1993). On the other hand, self-pollen deposition may be a selective mechanism contributing to the evolutionary diversity of floral forms (Barrett, 2002a). In self-incompatible species, genetic factors prevent self-fertilization by identifying and rejecting self-pollen (Hiscock and McInnis, 2003), similar to a lock-and-key mechanism (Wright and Barrett, 2010). Thus more than 100 families among angiosperms include self-incompatible species (Igic et al., 2008). As an example, approximately 40% of Solanaceae species, i.e., nearly 2,600 species, are self-incompatible (Whalen and Anderson, 1981; Igic et al., 2006). Further, Igic et al. (2008) estimated the frequency of self-incompatible species at approximately 40% in New World plant communities. This estimate was lower than the figure proposed by Wright and Barrett (2010) and Gibbs (2014), who reported that self-incompatible species accounted for half of all angiosperms. Self-pollen may result in ovule discounting in many self-incompatible species (Barrett, 2002a) and in a reduction in the number of cross-pollen tubes (Ockendon and Currah, 1977) and cross seeds (Kawagoe and Suzuki, 2005; Li et al., 2013). However, interference of self-pollen is absent in self-incompatible *Raphanus raphanistrum* (Brassicaceae) (Koelling and Karoly, 2007). The consequence of self-pollen deposition on stigma of self-incompatible flowers warrants further investigation.

In addition to solitary flowered plants, pollen discounting due to geitonogamy is common (Harder and Wilson, 1998). An earlier study showed that the flowers that received 1:1 and 2:1 self versus outcross pollination showed a lower seed set than those subjected to outcross pollination (Gibbs et al., 2004). However, in some self-incompatible species such as those of Bignoniaceae and Bombacaceae, self-pollen can overcome the self-sterility barrier and produce selfed seeds with the help of cross-pollen (Glendinning, 1960; Gribel et al., 1999). Thus, for example, Bertin and Sullivan (1988) revealed that mixed cross- and self-pollen produced fruits containing a certain proportion (2–33%) of selfed seeds, consistently with results reported for two bombacaceous species (Gribel et al., 1999; Gribel and Gibbs, 2002). However, the factors that determine the effect of self-pollen on female reproductive success and the response caused by self-pollen in self-incompatible species have not been fully explored. Additionally, in species that exhibit late-acting self-incompatibility (LSI), in which the pollen tube of the self-pollen can grow toward the ovules prior to the occurrence of rejection (Gibbs, 2014), the influence of self-pollen requires further research.

*Akebia quinata* Decaisne is a monecious LSI woody vine that produces large fleshy fruits that take 4 months to ripen. In this species, the growth rates of self-pollen tubes and outcross-pollen tubes shows no difference, and self-pollen tubes can reach the ovules but do not produce seeds (Kawagoe and Suzuki, 2005). An earlier study on *A. quinata* by Kawagoe and Suzuki (2003) showed that dimorphism—larger female flowers than male flowers—can prevent geitonogamy, and monoecy prevents sexual interference, at least in female flowers (Kawagoe and Suzuki, 2005). Earlier studies that explored the effect of self-pollen on female fitness in self-incompatible species, mostly used mixed pollination, pure selfing, pure crossing (Johnson et al., 2019), and hermaphrodite plants. However, the factors and specific effects of self-pollen on female reproductive success in monecious self-incompatible species have not been analyzed quantitatively. Here, we evaluated whether geitonogamy occurs in *A. quinata*, to understand the response of self-pollen in self-incompatible species in natural communities. Artificial pollination was performed to detect whether self-pollen affected female reproductive success under different factors, such as pollen numbers and different ratios of self- and cross-pollen loads. We used young fruit set of *A. quinata* to reflect the influence of self-pollen on female reproductive success to eliminate the interference of resource constraints. Specifically, we addressed the following questions: (1) Does the stigma of female flowers receive self-pollen from male flowers of the same plant? (2) What are the effects of self-pollen on female reproductive success and whether such effect depends on the intensity of self-pollen?

## Materials and methods

### Study species and sites

*Akebia quinata* has unisexual flowers that bloom from March to May in China. The inflorescences usually consist of 4–10 male flowers and 1–2 female flowers. A female flower usually has 4–10 divergent carpels, and each carpel has a stigma (Kawagoe and Suzuki, 2003). Approximately 200 ovules are present in each carpel. Female and male flowers have pale purple sepals and are usually structurally distinct, showing sexual dimorphism. Large plants can produce thousands of inflorescences and many male and female flowers open simultaneously (Kawagoe and Suzuki, 2002). Male flowers usually open 1–2 days later than female flowers, and the overlap between male and female flowering is approximately 1 week. Mucus is present on the stigma, which indicates the stigma receptivity. Our study was conducted at two sites: Jiufeng (30°52′2′′N, 114°50′48′′E), located in Wuhan (Hubei Province), and HongHua town (31°41′25′′N, 110°48′17′′E) in Shennongjia National Nature Reserve (Hubei Province). The study was conducted during the flowering seasons in 2016 and 2019.

### Tracking pollen movement

To examine whether geitonogamy occurs in the natural population, we tracked pollen movement in female flowers using a pollen dyeing experiment. Male flowers whose stamens did not dehisce on the day of the experiment were removed from the selected plant. Safranine aqueous solution (1%) was used to stain the pollen grains from the anthers of approximately 500 male flowers that were about to dehisce in that plant. A total of 89 stigmas from 20 female flowers from the same plant were harvested after the mucus on the stigma had dried. Pollen deposition per stigma was immediately observed under a light microscope (Nikon E100; Chiyoda, Japan). Pollen grains, including stained and natural pollen, were then counted. If a pollen grain on the stigma was stained, it must be from the same plant, which entailed that geitonogamy must occur.

### Stigmatic pollen loads under natural conditions

To examine the pollen load under natural pollination, we randomly selected 20 female flowers at Jiufeng. Pollen grain numbers on each stigma were counted under the light microscope.

### Hand-pollination experiments

To explore the effect of self-pollen on female reproductive success, we conducted hand-pollination with different self-and cross-pollen rates in approximately 13 populations. flowers were randomly selected in the 13 populations for each treatment to reduce the impact of individual variation on the results. The number of flowers and carpels used for hand-pollination in each treatment was recorded and showed in Table 1. A needle was used to collect the pollen from the anthers. Hand-pollination was performed using the pollen on the needle.

**TABLE 1:** The number of flowers and carpels applied to hand-pollination and young fruit in each treatment.

	C/S = 4:0	C/S = 3:1	C/S = 2:2	C/S = 1:3
**(a)Mixed pollen enough**				
Flower numbers applied to hand-pollination	52	56	58	51
Carpel numbers applied to hand-pollination	301	286	281	281
Young fruit numbers	143	163	80	17
**(b)Mixed pollen limit**				
Flower numbers applied to hand-pollination	47	47	52	50
Carpel numbers applied to hand-pollination	177	188	197	205
Young fruit numbers	103	68	50	22

C/S: ratio of cross- and self-pollen.

Mature buds were bagged before the stigma was covered by mucus. The cross-pollen grains used were from another population located more than 20 meters from the artificially pollinated plants. Experiments were conducted in two groups. The first group, called the pollen-limit group, consisted of stigmas with pollen grains that were lower than the approximate number of ovules. The second group, called the pollen-enough group, consisted of stigmas with pollen grains that were more than the approximate number of ovules. Each group was subjected to four treatments, cross-pollen: self-pollen (C/S) in ratios of 1:3, 2:2, 3:1, and 4:0 was deposited on the stigma. Hand-pollination was performed four times for each treatment. The number of pollen grains deposited on the stigma during each experiment was 34 ± 23 and 322 ± 69 in the pollen-limit group and pollen-enough group, respectively. Therefore, it was ensured that the total number of pollen grains in the pollen-limit group was less than the approximate number of ovules and that in the pollen-enough group was more than four times the approximate number of ovules. Self-pollen was deposited on half or two-thirds of the globose stigma and cross-pollen was applied to the rest of the stigma immediately. For each flower, young fruit set was calculated as young fruit number divided by the carpel number, and mature fruit set was calculated as mature fruit number divided by carpel number. Young fruit set was evaluated after 1 month of the artificial pollination. In turn, mature fruit set and seed set were evaluated 4 months after pollination, when mature fruits were harvested.

### Statistical analysis

Fruit set data were arcsine-transformed before analysis. Two-way analysis of variance (ANOVA) of a complete factorial test was conducted using JMP Pro 13.2 (SAS institute Inc., Cary, NC, United States) to investigate the effect of total pollen numbers and different C/S ratios on fruit and seed set. The total pollen numbers and C/S ratios were treated as fixed factors, while the carpel number was treated as a random factor. Multiple comparisons of the least squares means were then used to measure the effect of different C/S ratios and total pollen numbers on the fruit set. Seed set analysis under different treatments in the pollen-enough group was conducted using one-way ANOVA followed by the Tukey–Kramer honest significant difference test.

## Results

### Tracking pollen movement

The pollen dyeing experiment revealed that 65% of the flowers had dyed pollen grains on the stigma, out of which 25% and 30% had dyed pollen grains on all stigmas and on half of them. The remaining 10% had dyed pollen on less than half of the stigmas. These results indicated that geitonogamy occurred in *A. quinata* under natural conditions.

### Stigmatic pollen loads under natural conditions

Natural pollen deposition occurred on 73 stigmas of the 20 female flowers that were randomly collected at the Jiufeng location. The pollination rate of the flowers was 91.9%, and stigmas with deposition of more than 200 natural pollen grains accounted for 8.2% of all stigmas. In contrast, a large proportion (91.8%) of the stigmas had less than 200 pollen grains deposited on them, and there were (75.0%) on which all carpels were pollen limited.

### Fruit set and seed set

**FIGURE 1:**
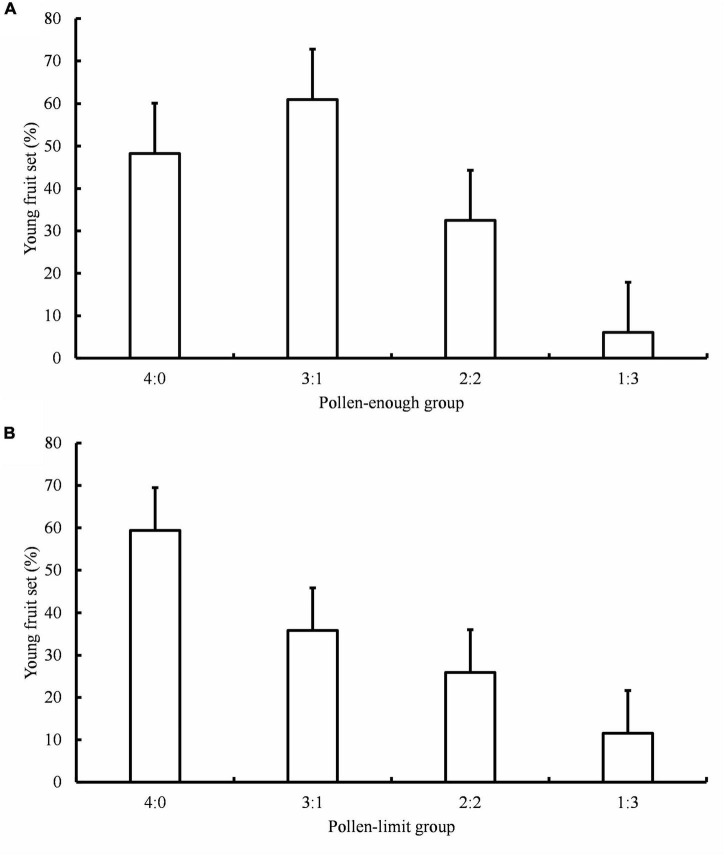
Young fruit set under different hand-pollination treatment. **(A)** Young fruit set of pollen-enough group; **(B)** Young fruit set of pollen-limit group; C/S: ratio of cross- and self-pollen. Data are presented as mean ± Standard Deviation (SD).

The carpel numbers and C/S ratios both affected the young fruit set in *A. quinata* (Table 2). Young fruit set did not differ significantly between C/S = 4:0 and C/S = 3:1 treatment in the pollen-enough group. However, it was significantly higher than the young fruit set results of C/S = 2:2 and C/S = 1:3 treatments (Figure 1; Table 3). Furthermore, the young fruit set of C/S = 2:2 was significantly higher than that of C/S = 1:3. Unexpectedly, there was no significant difference between the young fruit set of the pollen-enough group and the pollen-limit group (Figure 1; Tables 2, 3). The interactive effect of C/S and total mixed pollen numbers was analyzed and was shown to significantly affect the young fruit set (Table 2). The results showed that, except for the C/S = 3:1 treatment, which caused a significant difference if fruit set between the pollen-enough group and pollen-limit group, there were no significant differences among other pollination ratios (C/S = 4:0, 2:2 and C/S = 1:3) (Table 3).

**TABLE 2:** Results of two-way analysis of variance of a complete factorial test for the effect of deposition of different ratios of cross- and self-pollen, carpel numbers, and total pollen numbers on young fruit set.

Source of variation	DF	DDF	F-ratio	*P*-value
**(a) Fixed effect**				
C/S	3	404.4	37.937	<0.0001*
Total pollen numbers	1	398	1.691	0.1942
C/S and total mixed pollen numbers	3	404.4	3.423	**0.0173**
	**BLUP**	**SE**	**DDF**	**P**
**(b) Random effect**				
Carpel number	−0.152	0.029	278.5	<0.0001*

Fruit set data were arcsine-transformed, and mixed pollen numbers and different ratios of cross- and self-pollen were treated as fixed factors, while carpel number was treated as a random factor. C/S: ratio of cross- and self-pollen. Significant effects are denoted with an asterisk at P < 0.001.

**TABLE 3:** Multiple comparisons on young fruit set of the least squares means between different ratios of cross- and self-pollen and total pollen numbers.

(a) C/S	Least squares means
4:0	0.85^A^
3:1	0.743^A^
2:2	0.415^B^
1:3	0.184^C^
**(b) Total pollen numbers**	
Pollen-enough group	0.927^A^
Pollen-limit group	0.774^A^
**(c) C/S and total mixed pollen numbers**	
C/S = 3:1 and Pollen-enough	0.943^α^
C/S = 4:0 and Pollen-limit	0.906^α^
C/S = 4:0 and Pollen-enough	0.753^αβ^
C/S = 3:1 and Pollen-limit	0.570^βγ^
C/S = 2:2 and Pollen-enough	0.524^γ^
C/S = 2:2 and Pollen-limit	0.403^γ^
C/S = 1:3 and Pollen-limit	0.194^δ^
C/S = 1:3 and Pollen-enough	0.124^δ^

C/S: ratio of cross- and self-pollen. Differences in C/S are indicated by uppercase letters, whereas differences in total pollen numbers are indicated by lowercase letters. Significant effects are denoted at P < 0.05.

A total of 42 mature fruits were harvested from the pollen-enough group after successful hand-pollination. Conversely, no mature fruits in the pollen-limit group. Mature fruit set was showed in Supplementary Figure 1. The number of fertilized seeds per mature fruit in the C/S = 4:0 treatment was 153.5 ± 17.2, which was greater than that in the C/S = 3:1 (123.6 ± 5.4) and C/S = 2:2 (134.7 ± 9.4) treatments (Table 4). However, seed number per mature fruit did not differ significantly (*P* = 0.167 > 0.05) between different C/S ratios in the pollen-enough group (Tables 4, 5).

**TABLE 4:** Results of Tukey–Kramer honest significant difference test for the effect of treatment with different ratios of cross- and self-pollen on seed numbers per mature fruit in the pollen-enough group.

C/S	N	Seed numbers
		
4:0	4	153.5 ± 17.2^A^
3:1	28	123.6 ± 5.4^A^
2:2	9	134.7 ± 9.4^A^
1:3	1	–

Data are presented as mean ± standard error (SE). C/S: ratio of cross- and self-pollen.

Differences in seed number at different C/S ratios are indicated by uppercase letters.

Significant effects are denoted at P < 0.05.

**TABLE 5:** Result of the analysis of variance for the effect of treatment with different ratios of cross- and self-pollen on seed numbers per mature fruit in the pollen-enough group.

	df	MS	F	*P*-value
C/S	3	1482.873	1.780	0.167
Error	38	832.944		
Total	41			

Pollination treatment was considered a fixed effect. C/S: ratio of cross- and self-pollen.

## Discussion

A monecious woody vine, *A. quinata* that exhibits LSI was used in our study, to quantify the factors that determine the effect of self-pollen on the reproductive success and the response of self-pollen in self-incompatible monecious plants. In general, the transfer of self-pollen significantly reduced young fruit set. Conversely, treatment with a little self-pollen combined with cross-pollen did not reduce young fruit production and together with the physiological mechanisms of self-incompatibility, it declined the negative effects of self-fertilization. Our study aimed to quantify the factors that determine the effect of self-pollen on the reproductive success and the response of self-pollen in self-incompatible monecious plants. Additionally, we used young fruit set to reflect the influence of self-pollen on female reproductive success of the vines that produce large fleshy fruits, which take 4 months to ripen. This helped us to better understand the effects of self-pollen on the female reproductive success of flowering plants.

### Carpel numbers, ratio of self- and cross-pollen, and the interactive effect of ratio of self- and cross-pollen and total mixed pollen numbers affect female reproductive success

In self-incompatible plants, geitonogamy can lead to self-pollination and inbreeding depression, resulting in loss of female function (Eckert and Barrett, 1994), and loss of male function due to the dispersal of pollen within the same plant, which can not complete the potential outcrossing (Harder and Barrett, 1995). In addition to the common negative impacts of self-pollen, inbreeding depression may play a significant role in the maintenance of habitat segregation between *Mimulus guttaus* and *Mimulus nudatus* (Toll et al., 2021). According to the results of the experiments, young fruit set was not affected by the total pollen grain numbers but was influenced by the C/S ratio. Additionally, the results showed that, in general, the transfer of self-pollen significantly lesion young fruit set, which, however, did not differ significantly between C/S = 4:0 and C/S = 3:1 treatments in the pollen-enough group. Furthermore, there was a significant difference between young fruit set for the C/S = 3:1 treatment in the pollen-enough group and the pollen-limit group, which implies a condition in which a little self-pollen, together with cross-pollen, did not reduce young fruit production either, together with the physiological mechanisms of self-incompatibility to reduce self-fertilization. Other benefits of self-fertilization include reproductive assurance (Darwin, 1876; Pannell and Barrett, 1998), improved colonization ability (Baker, 1955), and complete self-fertilizing while contributing outcross pollen (Fisher, 1914; Lloyd, 1979), which confirmed that the most common evolutionary transition in angiosperms is from outcrossing to selfing (Barrett, 2002b).

Similarly, we found that the carpel numbers affect young fruit set in plants with an apocarpous gynoecium. Similar results have been preciously reported, wherein it was shown that pistil numbers can affect seed set in *Liriodendron chinense* (Magnoliaceae) (Huang and Guo, 2002). In addition, the results of our study indicated that seed set in mature fruits was not affected by self-pollen. A similar result was found in *Pedicularis siphonantha* and *P. longiflora* (Orobanchaceae), in which case, seed production was not affected by pollen quantity when pollen grains were present in a sufficient amount (Yang et al., 2005).

### Fertilized ovules and pollen quantity limitation affect fruit set and seed set

Our results revealed that pollen quantity limitation on the stigma and resource allocation significantly affected reproduction in *A. quinata.* From our earlier results, mature fruit set under natural conditions was 1.2 ± 0.4% (We marked 188 female flowers for a total of 949 carpels from two populations, in all 789 female flowers), which was similar to the results of C/S = 4:0 (1.4 ± 0.7%) in pollen-enough group. As the results obtained in August showed that severe resource constraints had a significant negative impact on the eventual reproductive success of the population, and as we wished to investigate the effects of different amounts and combinations of self vs. cross pollen grains on the reproductive success of *A. quinata*, young fruit set was used to eliminate the interference of resource constraints. Therefore, the young fruit set may better reflect the effect of self-pollen on female reproductive success in *A. quinata*. Pollen limitation occurs in approximately 62–73% of all insect-pollinated species (Ashman et al., 2004; Garcia-Camacho and Totland, 2009), and approximately 60% of 258 species show significant pollen limitation (Burd, 1994). Furthermore, compared with self-compatible species, pollen limitation is most likely to occur in self-incompatible species (Larson and Barrett, 2000; Knight et al., 2005), whereby, the female reproductive success may be significantly reduced in these plants. Haig and Westoby (1988) and Brookes et al. (2008) showed that factors such as pollen deposition and resource allocation may limit fruit and seed production, especially in plants with numerous flowers that often flower in early spring, when pollinators are scarce (Wesselingh, 2010).

Additionally, we hypothesized that the formation of mature fruits was affected by the number of fertilized ovules of *A. quinata*, and that mature fruits would be produced only when more than half of the ovules were fertilized. If the number of fertilized ovules was less than half of the number of ovules, abandonment of these low-seed fruits may occur, and resources would be relocated to fruits with more seeds. This was consistent with the findings of Stephenson (1981), who demonstrated that a low fruit set may result in fruit abortion in some species. Many studies have shown a relationship between the amount of pollen deposition on the stigma and fruit formation (Stephenson et al., 1995). According to a study by Quesada et al. (2001) on *Pachira quinata*, another LSI species, showed that fruit set occurred only after receiving an average of 422 pollen grains (2.6:1 ovule per pollen) on the stigma, consistently with results of Brookes et al. (2008), who reported that less than half of the ovules in *Stylidium armeria* formed seeds under conditions of supplemental pollen and resources. These findings imply that flowers may contain extra ovules (Brookes et al., 2008), a strategy that may result in selective abortion according to offspring quality (Korbecka et al., 2002). Kawagoe and Suzuki (2005) reported that the self-pollen of *A. quinata* may interfere with the formation of outcross pollinated seeds, possibly because ovules occupied by self-pollen would no longer be used for outcrossing. Mixed pollination (i.e., self- and cross-pollen) by natural pollinators often resulted in low fruit set in *A. quinata* due to ovule discounting; a phenomenon that was also observed in *Ceiba pentandra* (Gribel et al., 1999), *C. chodatii* (Gibbs et al., 2004), *Pseudobombax munguba* (Gribel and Gibbs, 2002), and *Cyrtanthus breviflorus* (Vaughton et al., 2010).

### Adaptive significance of self-incompatible monecious species

*Akebia quinata*, a self-incompatible species, transmits only 50% of the genes to the next generation. However, adaptations must exist in these self-incompatible plants, considering that half of all flowering plant species are self-incompatible, i.e., they impede interspecific hybridization (Baack et al., 2015; Broz et al., 2017), thereby creating a pollen–pistil barrier (Tovar-Méndez et al., 2014), and causing reproductive isolation (Baack et al., 2015). According to Wright and Barrett (2010), self-incompatibility may have some macroevolutionary advantage, allowing it to be maintained by species selection.

Self-fertilization is one of the most important selective forces that shapes floral evolution (Li et al., 2013). However, species that shift their mating systems toward greater selfing rates due to interspecific reproductive interference may face greater selective pressure than more outcrossing species. This is because the competitive ability of self-pollen is considered to be weaker than that of outcrossing pollen (Moreira-Hernández and Muchhala, 2019). Meanwhile, in self-fertilizing flowering plants, selfing may function as an effective barrier that mediates reproductive isolation (Brys et al., 2014; Castro et al., 2020). Selfing may increase post-zygotic costs (i.e., reduce hybrid vigor), following hybridization, thus preventing interspecific gene flow (Brys et al., 2016). Our results showed that self-pollen does not always interfere with female function, furthermore, female reproductive success depends on different factors. Our study showed the conditions under which self-pollen could or could not interfere with fruit and seed set in self-incompatible plants. Therefore, our results may provide a new perspective for explaining the effects of self-pollen on the female reproductive success in flowering plants and the adaptive significance of monecious self-incompatible species.

## Data availability statement

The original contributions presented in this study are included in the article/Supplementary material, further inquiries can be directed to the corresponding author.

## Author contributions

C-HW and X-FW conceived the idea and designed the research. C-HW, T-TZ, and W-QL performed the experiments and conducted fieldwork. C-HW analyzed the data. C-HW and T-TZ created the data figures and illustrations. C-HW, T-TZ, and X-FW wrote the manuscript. All authors contributed to the article and approved the submitted version.
