# Supplementary figures and images for: The influence of self-pollen deposition on female reproductive success in a self-incompatible plant, Akebia quinata
Source: Front Plant Sci. 2022 Aug 10;13:935217. doi: 10.3389/fpls.2022.935217 (PMC9399832; doi:10.3389/fpls.2022.935217)

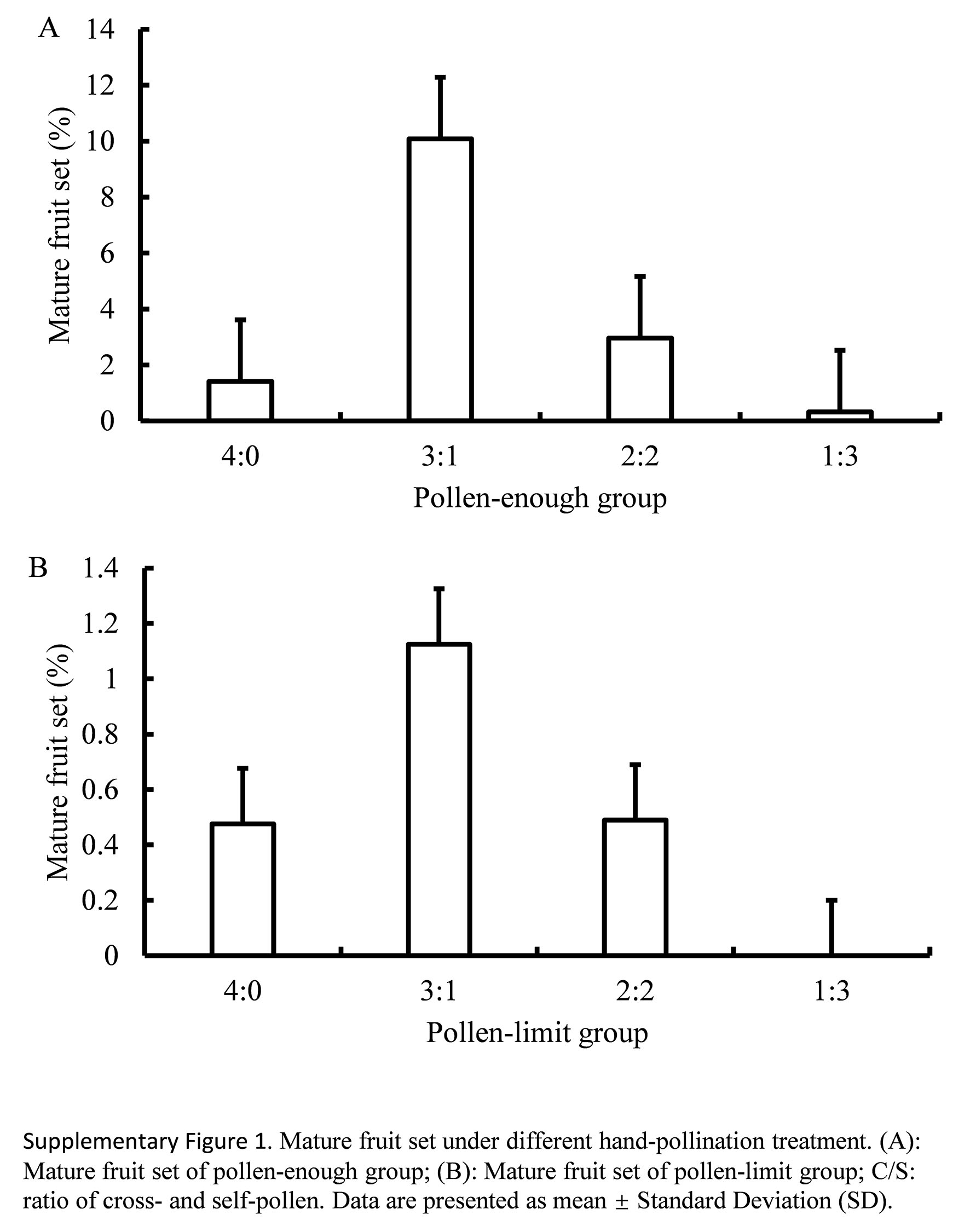

Supplement: Supplementary file 1 [file Image_1.jpg]
